# Phase-Specific and Lifetime Costs of Multiple Myeloma Among Older Adults in the US

**DOI:** 10.1001/jamanetworkopen.2021.16357

**Published:** 2021-07-09

**Authors:** Kaustuv Bhattacharya, John P. Bentley, Sujith Ramachandran, Yunhee Chang, Benjamin F. Banahan, Ruchit Shah, Nickhill Bhakta, Yi Yang

**Affiliations:** 1 Center for Pharmaceutical Marketing and Management, University of Mississippi School of Pharmacy, University; 2 Department of Pharmacy Administration, University of Mississippi School of Pharmacy, University; 3 Department of Nutrition and Hospitality Management, University of Mississippi, University; 4 Pharmerit International, Bethesda, Maryland; 5 Department of Global Pediatric Medicine, St Jude Children’s Research Hospital, Memphis, Tennessee

## Abstract

**Importance:**

Health care costs associated with diagnosis and care among older adults with multiple myeloma (MM) are substantial, with cost of care and the factors involved differing across various phases of the disease care continuum, yet little is known about cost of care attributable to MM from a Medicare perspective.

**Objective:**

To estimate incremental phase-specific and lifetime costs and cost drivers among older adults with MM enrolled in fee-for-service Medicare.

**Design, Setting, and Participants:**

A retrospective cohort study was conducted using population-based registry data from the 2007-2015 Surveillance, Epidemiology, and End Results database linked with 2006-2016 Medicare administrative claims data. Data analysis included 4533 patients with newly diagnosed MM and 4533 matched noncancer Medicare beneficiaries from a 5% sample of Medicare to assess incremental MM lifetime and phase-specific costs (prediagnosis, initial care, continuing care, and terminal care) and factors associated with phase-specific incremental MM costs. The study was conducted from June 1, 2019, to April 30, 2021.

**Main Outcomes and Measures:**

Incremental MM costs were calculated for the disease lifetime and the following 4 phases of care: prediagnosis, initial, continuing care, and terminal.

**Results:**

Of the 4533 patients with MM included in the study, 2374 were women (52.4%), 3418 (75.4%) were White, and mean (SD) age was 75.8 (6.8) years (2313 [51.0%] aged ≥75 years). The characteristics of the control group were similar; however, mean (SD) age was 74.2 (8.8) years (2839 [62.6%] aged ≤74 years). Mean adjusted incremental MM lifetime costs were $184 495 (95% CI, $183 099-$185 968). Mean per member per month phase-specific incremental MM costs were estimated to be $1244 (95% CI, $1216-$1272) for the prediagnosis phase, $11 181 (95% CI, $11 052-$11 309) for the initial phase, $5634 (95% CI, $5577-$5694) for the continuing care phase, and $6280 (95% CI, $6248-$6314) for the terminal phase. Although inpatient and outpatient costs were estimated as the major cost drivers for the prediagnosis (inpatient, 55.8%; outpatient, 40.2%), initial care (inpatient, 38.1%; outpatient, 35.5%), and terminal (inpatient, 33.0%; outpatient, 34.6%) care phases, prescription drugs (44.9%) were the largest cost drivers in the continuing care phase.

**Conclusions and Relevance:**

The findings of this study suggest that there is substantial burden to Medicare associated with diagnosis and care among older adults with MM, and the cost of care and cost drivers vary across different phases of the cancer care continuum. The study findings might aid policy discussions regarding MM care and coverage and help further the development of alternative payment models for MM, accounting for differential costs across various phases of the disease continuum and their drivers.

## Key Points

**Question:**

What are the incremental lifetime and phase-specific Medicare costs for older adults with multiple myeloma?

**Findings:**

In this cohort study of data from 4533 patients with multiple myeloma, the incremental lifetime multiple myeloma cost was $184 495, with the incremental monthly costs being the highest in initial and terminal care phases and lower in continuing care and prediagnosis phases. Although inpatient and outpatient care was the major cost driver in most phases, prescription drugs were the largest cost driver in the continuing care phase.

**Meaning:**

The findings of this study suggest that multiple myeloma care imposes a substantial economic burden to Medicare, with cost of care varying by phases of the disease continuum.

## Teaser

This cohort study examines the costs associated with the prediagnosis, initial care, continuing care, and terminal phases of multiple myeloma in older adults.

## Introduction

Cancer treatment poses a substantial financial burden to patients and their families, payers, and society in general. The estimated total direct medical costs attributable to cancer treatment in the US are more than $125 billion each year.^1^ Although multiple myeloma (MM) accounts for approximately 1.6% of annual incident cancer cases in the US,^2,3^ health care costs associated with MM treatment are higher than treatment costs of most other types of cancer.^4^ Costs associated with MM have increased greatly over the past decade, with outpatient costs, hospitalizations, and drug costs identified as factors associated with the observed increase.^5,6^ However, most of these studies have used a prevalence-based approach to estimate the cost of care associated with MM.

Cancer treatments differ as the disease progresses; therefore, treatment costs differ by each phase of cancer care.^7^ Extant literature^8,9,10,11,12^ suggests that health care expenditures across the entire cancer care continuum follow a U-shaped curve, with greater expenditure incurred at the time of diagnosis (ie, the initial care phase) and death (the terminal phase), and lower expenditures incurred in the continuing care phase between the initial and terminal care phases. Compared with prevalent cost analyses, phase-based cost estimates, in combination with survival estimates, yield reliable estimates of long-term disease burden or disease lifetime costs.^8,9,10,11,12^

There is limited literature on disease lifetime costs among patients with MM. Most patients with MM in the US are diagnosed at age 65 years or older,^13^ and Medicare is the primary payer. With the introduction of several novel agents, the landscape of MM treatment has changed substantially over the past decade, making it necessary to assess the cost of MM from a Medicare perspective. This study assessed the phase-specific and disease lifetime costs of MM compared with a matched noncancer group and incremental MM costs for each phase of the disease among older adults newly diagnosed with MM who were enrolled in fee-for-service Medicare.

## Methods

### Data Source and Patient Cohort

This retrospective cohort study used the 2007-2015 Surveillance, Epidemiology, and End Results (SEER) registry data linked with 2006-2016 Medicare administrative claims data. The study was conducted from June 1, 2019, to April 30, 2021. All study procedures were approved by the University of Mississippi Institutional Review Board with a waiver of informed consent owing to restrospective nature of data, and the study followed the Strengthening the Reporting of Observational Studies in Epidemiology (STROBE) reporting guideline for cohort studies.^14^

The MM cohort included Medicare beneficiaries who entered the SEER registry from 2007 through 2015 and had a primary cancer diagnosis of MM. Patients with MM needed to be aged 66 years or older at the time of MM diagnosis and to have continuous enrollment in Medicare Parts A and B for 12 months before and 2 months after the MM diagnosis. They were also required to have continuous Part D enrollment for at least 3 months before and 2 months after the MM diagnosis or until death to be included in the study. Individuals without cancer were included from a 5% sample of Medicare in the noncancer group if they were aged 65 years or older and were continuously enrolled in Medicare Parts A, B, and D for at least 15 months between becoming eligible for Medicare and death or the end of follow-up. Patients were excluded from the study sample if they had an unknown MM diagnosis date, the MM diagnosis was made at the time of death, or they had a history of any cancer before the MM diagnosis. In addition, beneficiaries in both the MM and noncancer groups were excluded if they were enrolled in Medicare Advantage plans at any time during the study period owing to a lack of availability of reimbursement billing records for these beneficiaries.^15,16,17^

Medicare beneficiaries in the MM group who died during the study period were matched 1:1 to noncancer beneficiaries who died on date of death and length of continuous Medicare eligibility. Similarly, beneficiaries in the MM group who did not die were matched to beneficiaries in the noncancer group who did not die on the end of follow-up date (defined as loss of Medicare eligibility or end of study period, whichever was earlier) and length of continuous Medicare eligibility. This matching was done to ensure comparable cost accumulation patterns and similar follow-up time between the 2 groups.^7,18^ To compare outcomes between individuals in the MM group and their matched noncancer counterparts over the same follow-up period, beneficiaries in the noncancer group were assigned the MM diagnosis date of their corresponding matched MM beneficiaries as the index date.

### Outcome Measures

The outcome variable of interest was all-cause health care cost. All costs were measured from a Medicare perspective and adjusted to 2016 US dollars using the Medical Care Component of the Consumer Price Index.^19^ In addition to total health care cost, specific care-setting costs (inpatient, outpatient, prescription medication, and other, including durable medical equipment, home health agency, and hospice use) were calculated.

Study covariates assessed at baseline included age, sex, race, geographic region, rural/urban status, and comorbidity and disability status. Comorbidity was assessed using the Deyo adaptation of the Charlson Comorbidity Index.^20^ Disability status was calculated using a validated, claims-based algorithm during the 12-month period before the MM diagnosis.^21^ Race was categorized as White, African American, Hispanic, and Other, using information from the Medicare Denominator file for the noncancer group and the SEER Patient Entitlement and Diagnosis Summary File for the MM group. The "Other" group included beneficiaries who were identified as Asians, North American Natives, and Other/Unknown. Race was included as a covariate to account for racial disparities in health care expenditure.

To estimate phase-specific MM costs, MM care was divided into 4 phases: (1) prediagnosis (3 months before MM diagnosis),^22,23^ (2) initial care, (3) continuing care, and (4) terminal care.^8,12,24,25^ On calculation of monthly costs for the entire cohort, mean cost per phase was assessed and then monthly phase-specific costs were estimated by multiplying mean cost per phase by monthly survival probabilities.^18^ For each phase, cost attributable to MM was calculated using a net cost method by subtracting the costs incurred by beneficiaries in the noncancer group from those incurred by beneficiaries in the MM group.^7,8,25^

Beneficiaries who died during the follow-up period were first assigned to the terminal phase. Any remaining time was next assigned to the initial phase, and then to the continuing care phase. Beneficiaries who did not die in the follow-up period were first assigned to the initial phase and then to the continuing care phase.^18^ Lifetime costs were estimated by summing total costs across all the phases in which a beneficiary was included, with total cost for each phase being calculated by multiplying monthly phase-specific costs by the number of months the beneficiary was in that phase.^15,18^

### Statistical Analysis

Descriptive statistics were used to depict baseline patient characteristics and costs, and statistical comparisons between the MM and the noncancer groups were made using the Cochran-Mantel-Haenszel test. Duration of the initial phase and terminal care phases were determined using joinpoint regression.^26,27,28^ Log-transformed mean monthly all-cause health care costs for MM beneficiaries who died during the study period were modeled using heteroscedasticity-adjusted, autocorrelated error models to determine the duration of the initial and terminal care phases. Additional information on joinpoint regression analysis is presented in the eMethods in the Supplement. For unadjusted health care costs, Wilcoxon signed rank tests were used to test for differences between the 2 groups. All costs attributable to MM (phase-specific, lifetime, and cost drivers within each phase) were estimated, controlling for clinical and sociodemographic characteristics at baseline. Generalized linear models with log link and γ distribution were used to assess incremental MM costs. Recycled predictions were used to account for covariate imbalance.^29,30^ In addition, sensitivity analyses were conducted for all unadjusted and adjusted estimates of costs attributable to MM using the 8-month period before death as the terminal phase, based on results of the joinpoint regression analysis.

For the joinpoint regression analyses, 2-tailed tests with Bonferroni-corrected significance levels were used to account for multiple comparisons. For all other analyses, statistical significance was determined using a 2-sided level of α = .05. Joinpoint regression analyses were conducted using the Joinpoint Regression Program, version 4.9.0.0 (Statistical Research and Applications Branch, National Cancer Institute). All other statistical analyses were performed using SAS, version 9.4 (SAS Institute Inc) and Stata, version 15 (StataCorp LLC).

## Results

Of the 6151 patients with MM and the 111 736 noncancer beneficiaries eligible for the study, 4533 matched pairs of MM and noncancer beneficiaries were included for analysis (eFigure 1 in the Supplement). A total of 2374 patients with MM (52.4%) were women, 3418 (75.4%) were White, and mean (SD) age was 75.8 (6.8) years (2313 individuals [51.0%]) aged ≥75 years). The characteristics of the control group were similar; however, mean (SD) age was 74.2 (8.8) years (2839 individuals [62.6%] aged ≤74 years). Additional sociodemographic and clinical characteristics of Medicare beneficiaries with MM and matched noncancer beneficiaries are presented in Table 1.

**Table 1.** Clinical and Sociodemographic Characteristics of Older Adults With MM and Matched Noncancer Cohort

Characteristics	No. (%)		*P* value^a^
	MM group (n = 4533)	Noncancer group (n = 4533)	
Living in urban areas			
No	524 (11.6)	313 (6.9)	<.001
Yes	4009 (88.4)	4220 (93.1)	
Geographic region			
Northeast	954 (21.0)	768 (16.9)	<.001
South	1231 (27.2)	941 (20.8)	
Midwest	575 (12.7)	424 (9.4)	
West	1773 (39.1)	2400 (52.9)	
Sex			
Male	2159 (47.6)	1837 (40.5)	<.001
Female	2374 (52.4)	2696 (59.5)	
Race/ethnicity			
White	3418 (75.4)	3253 (71.8)	<.001
African American	663 (14.6)	332 (7.3)	
Hispanic	130 (2.9)	230 (5.1)	
Other^b^	322 (7.1)	718 (15.8)	
Charlson Comorbidity Index score			
0	1156 (25.5)	1567 (34.6)	<.001
1	964 (21.3)	937 (20.7)	
2	709 (15.6)	586 (12.9)	
≥3	1704 (37.6)	1443 (31.8)	
Age, y			
66-69	942 (20.8)	2046 (45.1)	<.001
70-74	1278 (28.2)	793 (17.5)	
75-79	983 (21.7)	504 (11.1)	
80-84	740 (16.3)	424 (9.4)	
≥85	590 (13.0)	766 (16.9)	
Disability status			
No	3668 (80.9)	3460 (76.3)	<.001
Yes	865 (19.1)	1073 (23.7)	
Death			
No	2460 (54.3)	2460 (54.3)	NA
Yes	2073 (45.7)	2073 (45.7)	

Abbreviations: MM, multiple myeloma; NA, not applicable.

^a^ Statistical comparisons between the MM and the noncancer groups were made using the Cochran-Mantel-Haenszel test.

^b^ This designation included Asian, North American Native, and Other/Unknown race/ethnicity categories.

As shown in the Figure, A, joinpoint regression analysis for identifying the duration of the initial phase found a statistically significant inflection point in monthly cost trends at month 4 (monthly percent change, −15.0%; 95% CI; –17.7% to –12.2%). In addition, joinpoint regression analysis for identifying the duration of the terminal phase (Figure, B) showed statistically significant inflection points in monthly trend costs at month 3 (monthly percent change, 20.8%; 95% CI, 17.3% -24.1%) and month 8 (monthly percent change, 6.9%; 95% CI, 5.4%-8.4%) before death. Based on these results, the duration of the initial and terminal phases was defined as the 4-month period after MM diagnosis and the 3-month period before death. Visualization of monthly costs using median as well as 25th and 75th percentile costs is presented in eFigure 2 and eFigure 3 in the Supplement.

**Figure.**
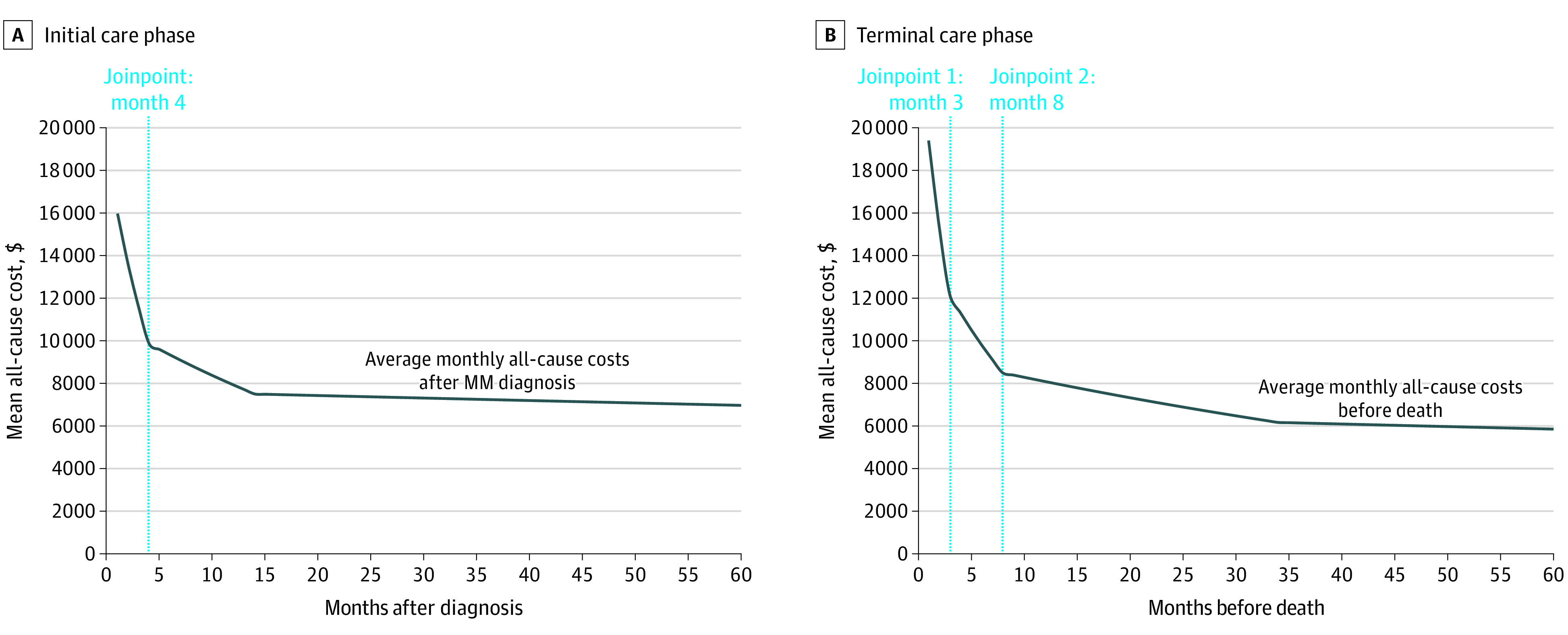
Joinpoint Regression for Identifying Duration of Care Duration of the initial (A) and terminal (B) care phases. MM indicates multiple myeloma.

Table 2 presents the results of the unadjusted analysis comparing costs between the MM group and the matched noncancer group. Beneficiaries in the MM group had significantly greater mean (SD) lifetime costs ($212 474.7 [$149 539.6] vs $54 085.6 [$78 265.3]) and per member per month cost in all the phases of care compared with their matched noncancer counterparts: prediagnosis, $2080.9 ($3971.0) vs $1566.8 ($4601.3); initial care, $10 384.5 ($9108.2) vs $1618.2 ($4177.1); continuing care, $6082.8 ($5582.0) vs $1495.0 ($3096.5); and terminal care, $14 417.0 ($11 572.1) vs $8853.1 ($13 575.5).

**Table 2.** Unadjusted Comparisons Between Older Adults With MM and Matched Noncancer Cohort

PMPM cost	MM group, $		Noncancer group, $		*P* value
	Mean (SD)	Median (IQR)	Mean (SD)	Median (IQR)	
Disease lifetime	212 474.7 (149 539.6)	184 160.1 (96 088.6-296 404.5)	54 085.6 (78 265.3)	24 734.6 (7078.1-69 988.3)	<.001
Prediagnosis phase	2080.9 (3971.0)	783.3 (361.3-1851.9)	1566.8 (4601.3)	353.3 (100.1-1019.1)	<.001
Outpatient	790.6 (1022.6)	475.5 (191.9-975.6)	395.6 (821.6)	107.5 (22.9-373.6)	<.001
Inpatient	896.0 (3244.4)	0.0 (0.0-0.0)	733.4 (3946.9)	0.0 (0.0-0.0)	<.001
Prescription drugs	292.2 (658.2)	139.7 (37.5-333.1)	268.6 (465.6)	117.5 (16.5-346.2)	.12
Other	102.1 (416.4)	0 (0.0-3.3)	169.3 (696.9)	0 (0.0-0.0)	.64
Initial care phase	10 384.5 (9108.2)	8724.8 (3119.5-14 595.3)	1618.2 (4177.1)	405.0 (115.6-1150.5)	<.001
Outpatient	3976.7 (3122.5)	3127.5 (1397.8-6035.8)	431.8 (953.1)	127.0 (28.5-415.9)	<.001
Inpatient	3685.2 (6936.0)	0.0 (0.0-4756.5)	692.5 (3365.9)	0.0 (0.0-0.0)	<.001
Prescription drugs, mean (SD)	2369.3 (3259.9)	427.7 (4366.5)	284.6 (590.0)	131.6 (342.8)	<.001
Other	353.3 (726.2)	5.1 (0.0-286.2)	209.4 (831.9)	0.0 (0.0-0.0)	<.001
Continuing care phase	6082.8 (5582.0)	4976.3 (2167.8-8620.5)	1495.0 (3096.5)	475.4 (159.1-1463.0)	<.001
Outpatient	1997.7 (2045.7)	1306.7 (587.6-2760.7)	383.2 (784.7)	168.4 (67.4-371.7)	<.001
Inpatient	1270.1 (3315.8)	317 (0.0-1388.5)	667.4 (2305.8)	0.0 (0.0-308.5)	<.001
Prescription drugs	2566.9 (3141.5)	1145.1 (212.5-4088.7)	237.1 (436.2)	110.1 (27.8-290.6)	<.001
Other	248.1 (655.7)	33.1 (0.0-192.0)	207.4 (690.1)	0.0 (0.0-67.0)	<.001
Terminal care phase^a^	14 417.0 (11 572.1)	11 889.3 (5933.3-19 255.1)	8853.1 (13 575.5)	4808.6 (1046.4-10 854.8)	<.001
Outpatient	3424.2 (3180.1)	2522.7 (1032.6-5031.1)	1404.0 (2021.7)	697.4 (118.9-1782.9)	<.001
Inpatient	7845.3 (9572.2)	5014.7 (0.0-10 971.5)	6128.9 (12 248.5)	0.0 (0.0-7490.6)	<.001
Prescription drugs	1846.8 (3207.5)	294.7 (86.9-2242.8)	331.2 (498.4)	174.1 (39.5-443.2)	<.001
Other	1300.8 (1606.8)	745.9 (64.6-1844.7)	989.0 (1641.4)	84.1 (0.0-1342.3)	<.001

Abbreviations: IQR, interquartile range; MM, multiple myeloma; PMPM, per member per month.

^a^ The 3-month period before death was considered the terminal phase.

Results of the adjusted analysis for assessing the incremental MM lifetime, phase-specific, and health care setting (inpatient, outpatient, prescription drug, and other) costs within each phase controlling for clinical and sociodemographic covariates are presented in Table 3. The mean adjusted lifetime cost for the MM group ($234 002; 95% CI, $232 232-$235 870) was significantly greater than that for the noncancer group ($49 507; 95% CI, $49 133-$49 903), with an incremental expenditure of $184 495 (95% CI, $183 099-$185 968).

**Table 3.** Multivariable Analysis Between Older Adults With MM and Matched Noncancer Cohort

PMPM cost	Mean estimate (95% CI), $		
	MM group	Noncancer group	Incremental MM cost
Disease lifetime	234 002 (232 232-235 870)	49 507 (49 133-49 903)	184 495 (183 099-185 968)
Prediagnosis phase	2589 (2531-2647)	1344 (1314-1375)	1244 (1216-1272)
Outpatient	873 (862-885)	365 (361-370)	508 (501-515)
Inpatient	1247 (1219-1278)	543 (523-565)	704 (696-713)
Prescription drugs	292 (289-295)	271 (268-273)	21 (21-21)
Other	182 (175-190)	153 (146-159)	29 (28-31)
Initial phase	12 557 (12 412-12 700)	1375 (1360-1391)	11 181 (11 052-11 309)
Outpatient	4369 (4336-4404)	396 (393-399)	3973 (3943-4005)
Inpatient	4821 (4736-4905)	546 (537-555)	4275 (4199-4350)
Prescription drugs	2508 (2493-2524)	270 (269-272)	2237 (2224-2252)
Other	868 (839-895)	147 (142-151)	721 (697-744)
Continuing care phase	6968 (6897-7042)	1334 (1320-1348)	5634 (5577-5694)
Outpatient	2166 (2149-2183)	364 (361-367)	1802 (1788-1816)
Inpatient	1645 (1623-1668)	558 (550-567)	1087 (1074-1101)
Prescription drugs	2751 (2736-2766)	225 (224-226)	2526 (2513-2540)
Other	410 (399-421)	196 (191-201)	214 (208-220)
Terminal phase	15 364 (15 286-15 447)	9084 (9038-9133)	6280 (6248-6314)
Outpatient	3699 (3677-3722)	1518 (1509-1528)	2181 (2168-2194)
Inpatient	8411 (8353-8472)	6351 (6307-6397)	2060 (2046-2075)
Prescription drugs	1947 (1938-1956)	346 (345-348)	1600 (1593-1608)
Other	1267 (1257-1276)	806 (800-812)	461 (457-464)

Abbreviations: MM, multiple myeloma; PMPM, per member per month.

The incremental MM costs (per member per month) were $1244 (95% CI, $1216-$1272) for the prediagnosis phase, $11 181 (95% CI, $11 052-$11 309) for the initial phase, $5634 (95% CI, $5577-$5694) for the continuing care phase, and $6280 (95% CI, $6248-$6314) for the terminal phase (Table 3). Inpatient services (55.8%), followed by outpatient services (40.2%), were the largest factors in incremental MM costs in the prediagnosis phase (eFigure 4 in the Supplement). In the initial care phase, the incremental MM costs were mostly associated with inpatient costs (38.1%) and outpatient costs (35.5%). Similar to the initial phase, incremental MM costs in the terminal care phase were associated with inpatient (33.0%) and outpatient costs (34.6%). However, in the continuing care phase, prescription drug costs were the largest factor (44.9%) involved in incremental MM costs. Sensitivity analysis produced similar results as the base case (eTable 1 and eTable 2 in the Supplement).

## Discussion

In this study, we used a phase-based costing approach to estimate incremental costs for each phase of MM care and identified factors associated with these phase-specific costs. To our knowledge, this is the first study to estimate the cost burden of MM from a Medicare perspective. Results from our study might provide the benchmark of MM costs and can aid evaluations of alternative payment models in MM.^31^

There is a substantial cost burden associated with MM care. Our study estimates the incremental lifetime cost of MM to be $184 495. This finding is consistent with previous research.^7^ With the approval of several anti-MM drugs since 2007 and the paradigm shift seen in MM care along with increased survival for patients with MM, our study presents a more current estimate of costs associated with MM care.

Most studies using a phase-based cost modeling approach have used the first 6 or 12 months after diagnosis as the initial phase and the 12 months before death as the terminal phase.^7,15,31,32,33,34,35^ This approach is generic and does not take into account the variations in care patterns for different cancers. Our study used a data-driven approach to identify the duration of the initial and terminal care phases for patients with MM enrolled in Medicare. This approach to identify duration of phases of cancer care has been used in previous studies.^26,36,37^

Our study estimated the incremental phase-specific costs of MM for each of the 4 phases of care—prediagnosis, initial, continuing care, and terminal—standardized in per member per month units. The incremental phase-specific costs were highest for the initial care phase, followed by the terminal phase, with costs being slightly lower for the continuing care phase and lowest for the prediagnosis phase. Our findings of cost variations between MM care phases are consistent with earlier research,^7^ with cost changes following a U-shaped curve: higher costs in the initial phase that then dipped in the continuing care phase before increasing again in the terminal phase. The initial care phase includes costs for further diagnostic tests and primary course of treatment, including surgery, chemotherapy, radiotherapy, and adjuvant therapy.^9,38^ The terminal care phase includes costs for intensive care services and palliative end-of-life care.^9,38^ We found that costs in both the initial and terminal care phases are associated mainly with hospitalizations and outpatient care. In contrast, the continuing care phase involves costs associated with lower-intensity maintenance therapy,^9,38^ with prescription drugs the primary cost drivers. This difference explains the higher costs in the initial and terminal care phases compared with the continuing care phase. Our findings are similar to those seen in studies of other cancer types that have assessed phase-specific costs.^9,26^

We also noted that inpatient and outpatient costs were the major cost drivers in all 4 phases of MM care. Prescription drug costs were a significant factor in the initial and terminal care phases and the biggest cost driver in the continuing care phase. Changes in cost drivers seen in our study are consistent with those of previous studies that assessed cost drivers among commercially insured patients with MM.^5,33^ Although it is understandable that hospitalization is one of the most expensive health care services, the high outpatient costs across all phases are potentially explained by the current treatment landscape in MM. Over the past decade, although new treatments have prolonged survival among patients with MM, the disease remains incurable, with MM care largely provided in the outpatient setting. Our finding that prescription drug costs represent the major cost driver in the continuing care phase is indicative of the long-term use of expensive immunotherapy drugs that are often used to prolong survival in patients.^31^

This study noted substantial economic burden associated with the diagnosis and treatment of MM among Medicare enrollees. Information on the phase-specific costs and cost drivers in each phase may help shape implementation of bundled payment models in MM.^39^ A recent analysis of cost of care for patients with MM under the episode-based oncology care model reported use of novel MM agents to be significantly associated with expenditures exceeding the expected target prices.^40^ Similar findings have been reported for other cancers.^41^ This report of incremental costs highlights the need for the oncology care model to be more flexible and account for the dynamic nature of cancer care along the disease continuum, especially novel agent use, for determining target price for episodes of care. Our study findings can inform future adjustments to the oncology care model specific to MM, adjusting for cost drivers at various phases of the care continuum.

### Strengths and Limitations

This study has several strengths. First, it used a data-driven approach to identify duration of various phases of MM care, making the study results relevant to patients with MM managed in real-world clinical settings. Second, owing to the high accuracy of the SEER registry data,^42^ capturing the MM diagnosis in our study is more accurate compared with studies using claims-based algorithms. Third, because most patients with newly diagnosed MM are aged 65 years or older and Medicare is the primary payer for these patients, the lifetime and phase-specific cost estimates provide an estimate of MM care burden to Medicare.

The study has limitations. Similar to other administrative claims-based studies, billing coding errors may bias our study estimates. The study findings are limited to patients with MM enrolled in fee-for-service Medicare who were aged 66 years or older at the time of MM diagnosis. In addition, given the study design, the comparator noncancer group may be sicker than the average Medicare fee-for-service population without cancer. However, our choice of the comparator group allows us to draw reasonable estimations about incremental costs attributable to MM. Furthermore, to account for differences in the health and composition of the control group, we used statistical methods that allowed us to account for confounders in the analysis. Future studies may consider comparing costs for MM beneficiaries with the average Medicare beneficiaries or other cancer types to understand the public health impact. This study also used data to identify the duration of MM care phases. This approach should be substantiated with clinical knowledge to determine the duration of care phases while reporting costs from MM diagnosis to death. Moreover, examination of patient-level characteristics associated with aggressive care and patient-level drivers of phase-specific costs, such as the terminal phase, was beyond the scope of this study and should be examined in future research.

## Conclusions

Our study used a data-driven approach to identify the duration of initial and terminal phases of care along the MM care continuum and highlighted the substantial economic burden associated with MM diagnosis and care. Findings on the economic burden of MM, including its lifetime and phase-specific costs, and cost drivers in each phase of MM care can aid policy discussions regarding MM care and coverage. The findings of this study can also be used to help the development of bundled payment models specific to MM, adjusting for costs across various phases of the disease continuum and their associated factors.

## Notes

Supplement. **eMethods.** Description of Joinpoint Regression Analysis **eTable 1.** Sensitivity Analysis Results of Unadjusted Comparisons Between Older Adults With Newly Diagnosed Multiple Myeloma (MM) and Non-cancer Medicare Beneficiaries on Lifetime Costs and Phase-Specific Costs – Considering the 8-Month Period Prior to Death as the Terminal Phase, 2007-2016 **eTable 2.** Sensitivity Analysis - Multivariable Analysis Between Older Adults With Newly Diagnosed Multiple Myeloma (MM) and Non-Cancer Medicare Beneficiaries on Disease Lifetime and Phase-Specific Costs (8-Month Terminal Phase), 2007-2016 **eFigure 1.** Study Flow Diagram **eFigure 2.** Visualization of Monthly All-Cause Cost Post-Diagnosis: Mean, Median, 25th Percentile, and 75th Percentile **eFigure 3.** Visualization of Monthly All-Cause Cost Prior to Death: Mean, Median, 25th Percentile, 75th Percentile **eFigure 4.** Drivers of Adjusted Incremental Multiple Myeloma (MM) Costs by Phases of Care
