# Supplementary material for: Phase-Specific and Lifetime Costs of Multiple Myeloma Among Older Adults in the US
Source: JAMA Netw Open. 2021 Jul 9;4(7):e2116357. doi: 10.1001/jamanetworkopen.2021.16357 (PMC8271356; doi:10.1001/jamanetworkopen.2021.16357)
Supplement: Supplement. — eMethods. Description of Joinpoint Regression Analysis eTable 1. Sensitivity Analysis Results of Unadjusted Comparisons Between Older Adults With Newly Diagnosed Multiple Myeloma (MM) and Non-cancer Medicare Beneficiaries on Lifetime Costs and Phase-Specific Costs – Considering the 8-Month Period Prior to Death as the Terminal Phase, 2007-2016 eTable 2. Sensitivity Analysis - Multivariable Analysis Between Older Adults With Newly Diagnosed Multiple Myeloma (MM) and Non-Cancer Medicare Beneficiaries on Disease Lifetime and Phase-Specific Costs (8-Month Terminal Phase), 2007-2016 eFigure 1. Study Flow Diagram eFigure 2. Visualization of Monthly All-Cause Cost Post-Diagnosis: Mean, Median, 25th Percentile, and 75th Percentile eFigure 3. Visualization of Monthly All-Cause Cost Prior to Death: Mean, Median, 25th Percentile, 75th Percentile eFigure 4. Drivers of Adjusted Incremental Multiple Myeloma (MM) Costs by Phases of Care [file jamanetwopen-e2116357-s001.pdf]

Supplemental Online Content

Bhattacharya K, Bentley JP, Ramachandran S, et al. Phase-specific and lifetime costs of multiple myeloma among older adults in the US. *JAMA Netw Open*. 2021;4(7):e2116357. doi:10.1001/jamanetworkopen.2021.16357

eMethods. Description of Joinpoint Regression Analysis
eTable 1. Sensitivity Analysis Results of Unadjusted Comparisons Between Older Adults With Newly Diagnosed Multiple Myeloma (MM) and Non-cancer Medicare Beneficiaries on Lifetime Costs and Phase-Specific Costs – Considering the 8-Month Period Prior to Death as the Terminal Phase, 2007-2016
eTable 2. Sensitivity Analysis - Multivariable Analysis Between Older Adults With Newly Diagnosed Multiple Myeloma (MM) and Non-Cancer Medicare Beneficiaries on Disease Lifetime and Phase-Specific Costs (8-Month Terminal Phase), 2007-2016
eFigure 1. Study Flow Diagram
eFigure 2. Visualization of Monthly All-Cause Cost Post-Diagnosis: Mean, Median, 25th Percentile, and 75th Percentile
eFigure 3. Visualization of Monthly All-Cause Cost Prior to Death: Mean, Median, 25th Percentile, 75th Percentile
eFigure 4. Drivers of Adjusted Incremental Multiple Myeloma (MM) Costs by Phases of Care

This supplemental material has been provided by the authors to give readers additional information about their work.

© 2021 Bhattacharya K et al. *JAMA Network Open*.

eMethods

Joinpoint regression analysis

Joinpoint regression is a piecewise linear regression used to identify the best-fitting points where statistically significant changes in the trends of monthly costs occur.[1,2] Separate models were estimated for the initial phase and the terminal phase. The model selection parameters for the joinpoint regression analyses were based on diagnostic tests for heteroscedasticity – using the Cook-Weisberg test for heteroscedasticity[3], and autocorrelation - using the Breusch-Godfrey LM test for autocorrelation[4,5]. Based on results for tests of heteroscedasticity and autocorrelation, heteroscedasticity-adjusted, autocorrelated error models were selected. Due to the skewed nature of cost data, log-transformed average monthly costs were modeled to detect statistically significant points of inflection in the trends of monthly costs. For the initial phase model, average monthly costs were modeled from MM diagnosis to death, and for the terminal phase model, costs were modeled backward from death to MM diagnosis. This study used a minimum of 0 joinpoints to a maximum of 5 joinpoints to identify best fit of data using the grid search method[2] for each joinpoint regression model. Joinpoints were defined as points of inflection in the trend of monthly costs. The joinpoints were then used to estimate the duration of the initial and terminal phases.

The sequential grid search algorithm initially tests for H_0: kmin (minimum – 0 in our case) joinpoints against H_1: kmax (maximum – 5 in our case) joinpoints.[2] If the null is rejected, it runs permutation tests increasing kmin by 1. If not, permutation tests are run by decreasing kmax by 1. This sequential grid search process continues until the final permutation test for H_0: k joinpoints against H_1: (k + 1) joinpoints, where $\text{kmin} \leq k < \text{kmax}$.[2] The final model estimates

© 2021 Bhattacharya K et al. *JAMA Network Open*.

existence of (k+1) joinpoints if the null hypothesis for the final model is rejected. If it fails to reject the null hypothesis for the final model, an estimated k joinpoints are considered. Since multiple comparisons are made to determine the number of joinpoints for the final model, Bonferroni-corrected significance levels are used for each individual test.[2] Joinpoint regression analyses were conducted using the Joinpoint Regression Program, Version 4.9.0.0 (Statistical Research and Applications Branch, National Cancer Institute).

© 2021 Bhattacharya K et al. *JAMA Network Open*.

eFigure 1: Study Flow Diagram

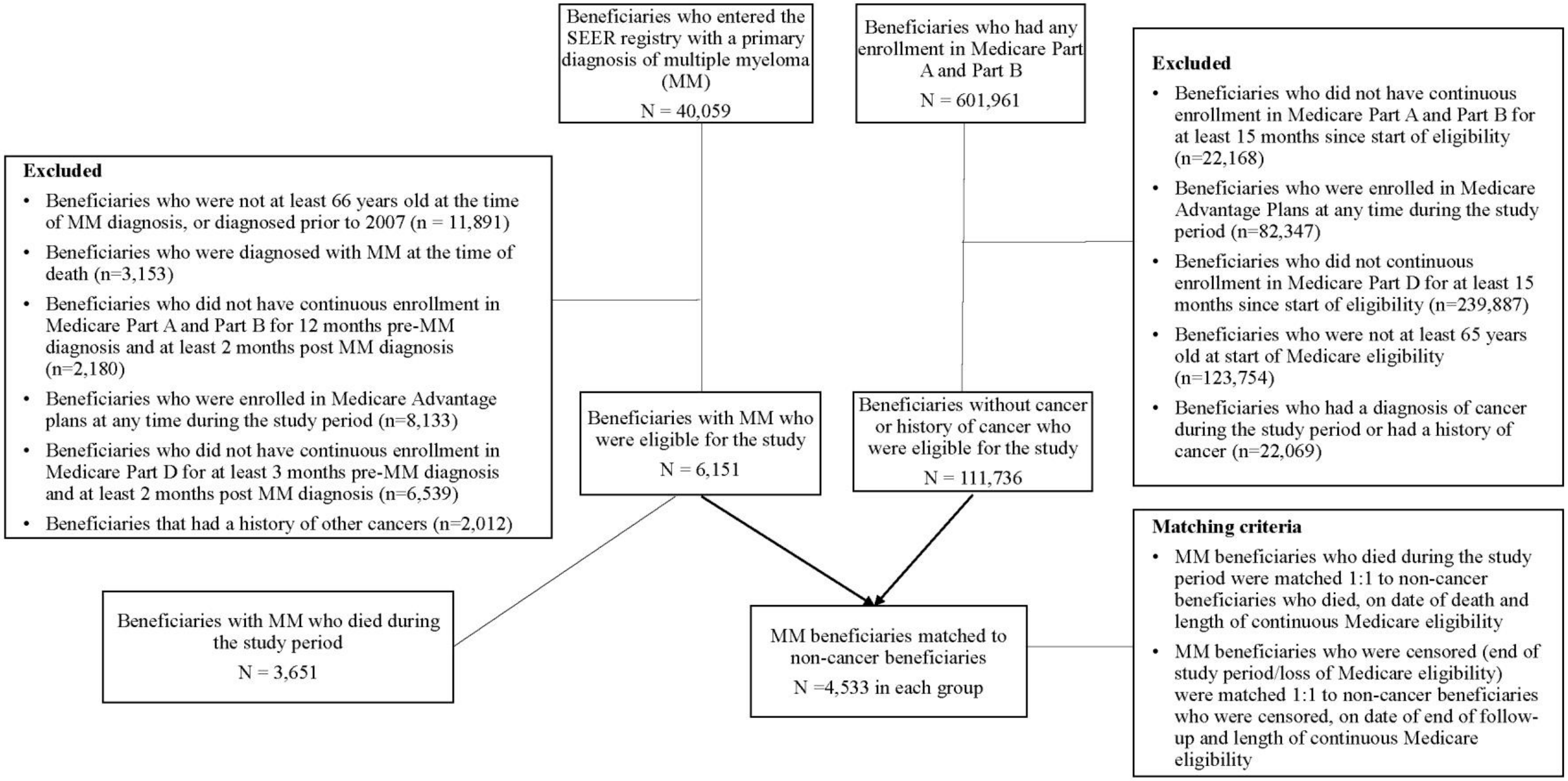

© 2021 Bhattacharya K et al. *JAMA Network Open*.

eFigure 2: Visualization of monthly all-cause cost post-diagnosis: mean, median, 25th percentile, and 75th percentile

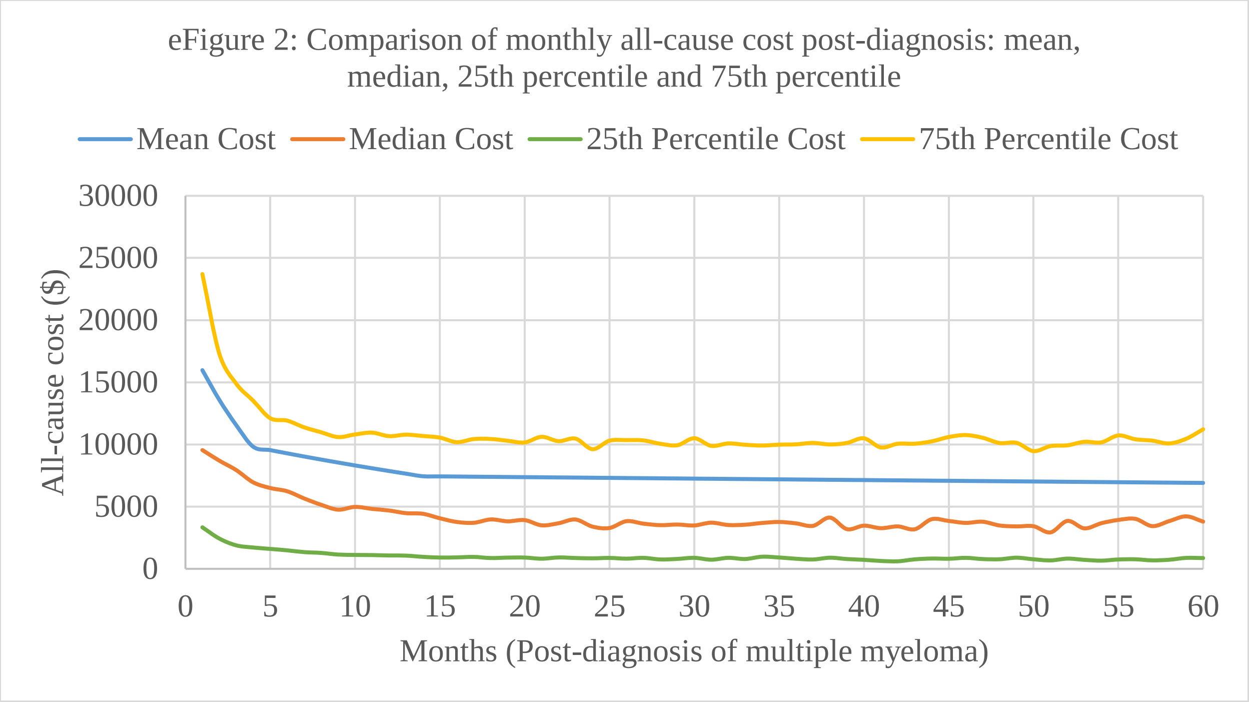

eFigure 3: Visualization of monthly all-cause cost prior to death: mean, median, 25th percentile, 75th percentile

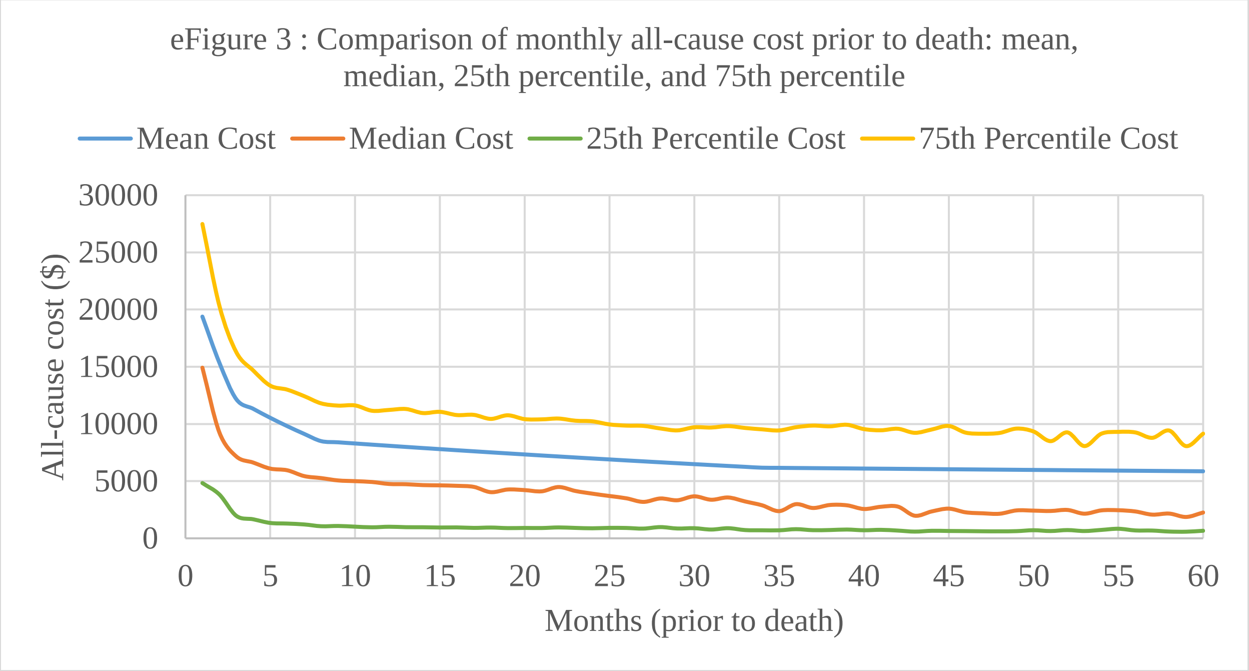

© 2021 Bhattacharya K et al. *JAMA Network Open*.

eFigure 4: Drivers of adjusted incremental multiple myeloma (MM) costs by phases of care

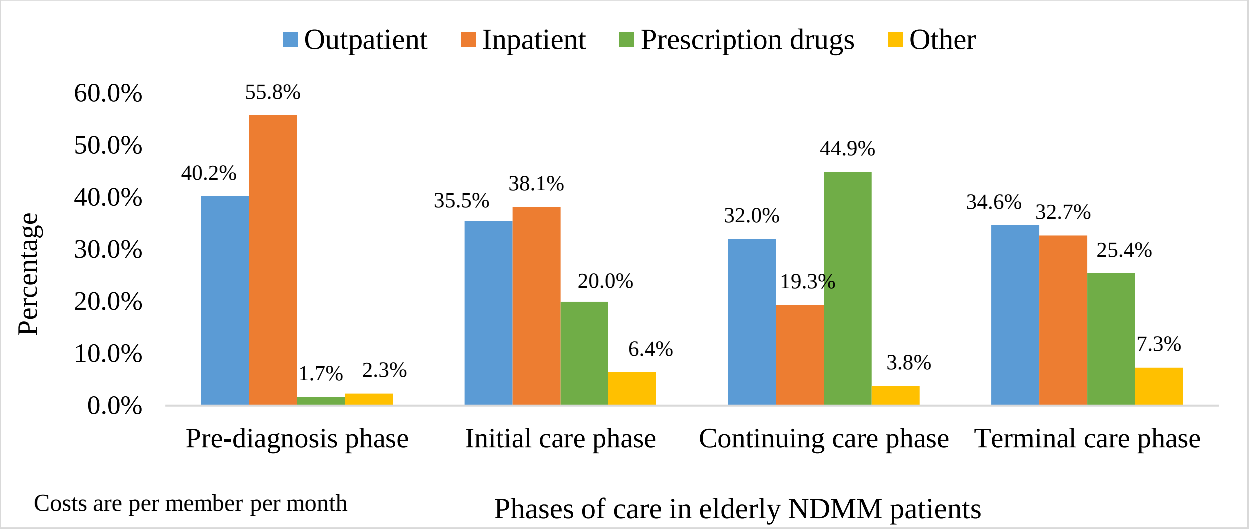

© 2021 Bhattacharya K et al. *JAMA Network Open*.

eTable 1 – Sensitivity Analysis results of unadjusted comparisons between older adults with newly diagnosed multiple myeloma (MM) and non-cancer Medicare beneficiaries on lifetime costs, and phase-specific costs, 2007-2016**

Cost Type (PMPM)	MM group (in US$)		Non-cancer group (in US$)		p
	Mean (SD)	Median (IQR)	Mean (SD)	Median (IQR)	
Disease Lifetime	217,039.2 (153,819.4)	186,917.8 (206,507.9)	56,008 (81,928.1)	25,098.1 (64,482.8)	<0.001
Pre-diagnosis Phase	2,080.9 (3,971.0)	783.3 (1,490.6)	1,566.8 (4,601.3)	353.3 (919.0)	<0.001
Outpatient	790.6 (1,022.6)	475.5 (783.7)	395.6 (821.6)	107.5 (350.7)	<0.001
Inpatient	896.0 (3,244.4)	0.0 (0.0)	733.4 (3,946.9)	0.0 (0.0)	<0.001
Prescription drugs	292.2 (658.2)	139.7 (295.6)	268.6 (465.6)	117.5 (329.7)	0.124
Other	102.1 (416.4)	0.0 (3.3)	169.3 (696.9)	0.0 (0.0)	0.643
Initial Care Phase	10,023.4 (8,591.0)	8,517.0 (11,290.8)	1,443.5 (4,362.3)	374.3 (903.6)	<0.001
Outpatient	3,962.4 (3,106.7)	3,094.7 (4,670.1)	399.6 (885.0)	119.1 (368.8)	<0.001
Inpatient	3,306.9 (6,276.3)	0.0 (4,321.3)	606.8 (3,616.4)	0.0 (0.0)	<0.001
Prescription drugs	2,434.6 (3,308.0)	441.2 (4,512.3)	274.7 (594.4)	127.2 (328.4)	<0.001
Other	319.6 (668.7)	0.0 (207.4)	162.5 (696.8)	0.0 (0.0)	<0.001
Continuing Care Phase	5,787.4 (4,848.5)	4,719.1 (6,451.1)	1,220.9 (2,539.2)	417.9 (1,068.9)	<0.001
Outpatient	1,933.3 (1,955.3)	1,249.4 (2,098.7)	344.7 (709.6)	158.3 (278.2)	<0.001
Inpatient	1,039.8 (2,311.9)	234.7 (1,162.0)	487.3 (1,792.8)	0.0 (199.3)	<0.001
Prescription drugs	2,607.4 (3,149.6)	1,163.8 (3,978.3)	230.7 (444.1)	103.8 (250.2)	<0.001
Other	206.9 (584.7)	25.2 (151.0)	158.1 (569.4)	0.0 (40.4)	<0.001
Terminal Care Phase	11,685.1 (8,243.3)	10,160.3 (9,984.7)	5,919.0 (7,431.9)	3,674.1 (6,332.5)	<0.001
Outpatient	3,241.3 (2,739.2)	2,490.3 (3,525.3)	1,061.7 (1,529.4)	539.0 (1,086.9)	<0.001
Inpatient	5,354.1 (6,263.5)	3,456.4 (6,034.6)	3,679.0 (6,397.3)	1,199.5 (4,773.2)	<0.001
Prescription drugs	2,205.7 (3,153.8)	506.2 (3,105.8)	374.4 (480.3)	234.5 (440.8)	<0.001
Other	883.9 (1,155.4)	492.7 (1,064.3)	803.9 (1,386.1)	124.6 (924.7)	<0.001

** Considering the 8-month period prior to death as the terminal phase;
PMPM – per member per month ; SD - standard deviation; IQR - interquartile range

© 2021 Bhattacharya K et al. *JAMA Network Open*.

eTable 2 - Multivariable analysis between older adults with newly diagnosed multiple myeloma (MM) and non-cancer Medicare beneficiaries on disease lifetime and phase-specific costs (8-month terminal phase), 2007-2016

Cost Type (PMPM)	MM group (mean, in US$)			Non-cancer group (mean, in US$)			Incremental MM cost (mean, in US$)		
	Estimate	95% LCL	95% UCL	Estimate	95% LCL	95% UCL	Estimate	95% LCL	95% UCL
Disease Lifetime	238,860	237,049	240,767	51,313	50,924	51,722	187,548	186,126	189,045
Pre-diagnosis Phase	2,589	2,531	2,647	1,344	1,314	1,375	1,244	1,216	1,272
Outpatient	873	862	885	365	361	370	508	501	515
Inpatient	1,247	1,219	1,278	543	523	565	704	696	713
Prescription drugs	292	289	295	271	268	273	21	21	21
Other	182	175	190	153	146	159	29	28	31
Initial Phase	12,179	12,045	12,314	1,283	1,268	1,297	10,897	10,777	11,017
Outpatient	4,354	4,323	4,389	376	373	379	3,979	3,950	4,010
Inpatient	4,378	4,301	4,454	504	495	512	3,875	3,805	3,942
Prescription drugs	2,584	2,568	2,601	264	262	266	2,320	2,306	2,335
Other	875	846	903	122	118	126	753	728	777
Continuing Care Phase	6,598	6,527	6,669	1,092	1,080	1,103	5,506	5,447	5,566
Outpatient	2,106	2,090	2,123	333	330	335	1,774	1,760	1,788
Inpatient	1,322	1,302	1,343	352	345	359	970	957	984
Prescription drugs	2,830	2,814	2,847	219	218	220	2,612	2,597	2,627
Other	352	343	361	156	152	160	196	191	201
Terminal Phase	12,363	12,304	12,422	5,928	5,900	5,957	6,435	6,404	6,466
Outpatient	3,482	3,461	3,503	1,108	1,102	1,115	2,374	2,359	2,388
Inpatient	5,721	5,683	5,759	3,793	3,768	3,817	1,928	1,915	1,942
Prescription drugs	2,315	2,305	2,326	393	391	395	1,922	1,914	1,931
Other	853	845	860	617	611	622	236	234	238
LCL – lower confidence limit; UCL – upper confidence limit; PMPM – per member per month									

© 2021 Bhattacharya K et al. *JAMA Network Open*.

References

1. Atkins M, Coutinho AD, Nunna S, Gupte-Singh K, Eaddy M. Confirming the timing of phase-based costing in oncology studies: a case example in advanced melanoma. *Journal of Medical Economics*. 2018;21(2):212-217. doi:10.1080/13696998.2017.1391818

2. Kim H-J, Fay MP, Feuer EJ, Midthune DN. Permutation tests for joinpoint regression with applications to cancer rates. *Statistics in Medicine*. 2000;19(3):335-351.

3. Cook RD, Weisberg S. Diagnostics for heteroscedasticity in regression. *Biometrika*. 1983;70(1):1-10.

4. Breusch TS. Testing for autocorrelation in dynamic linear models. *Australian Economic Papers*. 1978;17(31):334-355.

5. Godfrey LG. Testing against general autoregressive and moving average error models when the regressors include lagged dependent variables. *Econometrica: Journal of the Econometric Society*. Published online 1978:1293-1301.

© 2021 Bhattacharya K et al. *JAMA Network Open*.
